# Misperceptions and barriers to obesity management: Italian data from the ACTION-IO study

**DOI:** 10.1007/s40519-020-00907-6

**Published:** 2020-05-08

**Authors:** Paolo Sbraccia, Luca Busetto, Ferruccio Santini, Mariarosaria Mancuso, Paolo Nicoziani, Antonio Nicolucci

**Affiliations:** 1 Internal Medicine Unit and Obesity Center, Department of Systems Medicine, University of Rome Tor Vergata, Via Montpellier 1, 00133 Rome, Italy; 2 Center for the Study and the Integrated Treatment of Obesity, University Hospital of Padua, Padua, Italy; 3 Obesity and Lipodystrophy Center, Endocrinology Unit, University Hospital of Pisa, Pisa, Italy; 4 Novo Nordisk SpA, Rome, Italy; 5 Center for Outcomes Research and Clinical Epidemiology, Pescara, Italy

**Keywords:** Obesity, Obesity care, Healthcare professionals, Italy, Perception, Attitude

## Abstract

**Purpose:**

Despite the increasing prevalence of obesity in Italy, it remains largely underdiagnosed and undertreated. We aimed to identify the perceptions, attitudes, behaviours and barriers to effective obesity care among people with obesity (PwO) and healthcare professionals (HCPs) in Italy.

**Methods:**

The ACTION-IO study was an online cross-sectional survey conducted in 11 countries from June to October 2018. Findings from the Italian cohort are reported here.

**Results:**

The survey was completed by 1501 PwO and 302 HCPs in Italy. Most PwO (84%) and HCPs (77%) acknowledged the large impact of obesity on overall health. However, fewer PwO (62%) than HCPs (91%) perceived obesity as a chronic disease. Most PwO (84%) assumed full responsibility for their weight loss. A median of 3 (mean 6) years elapsed between when PwO started struggling with obesity and when they first discussed their weight with an HCP. Many PwO expressed that they liked (80%) or would like (74%) their HCPs to initiate weight management conversations, and only 3% were offended by such a conversation. For 77% of HCPs, perceiving their patients as unmotivated or disinterested in losing weight prevented them from initiating these conversations. Short appointment times were also considered a limiting factor for 40% of HCPs.

**Conclusions:**

Most PwO took complete responsibility for their own weight loss and waited considerable time before seeking help from an HCP. There is a need for improved education of both PwO and HCPs and for a more positive attitude from HCPs towards initiating weight discussions with PwO.

*Trial registration* ClinicalTrials.gov: Awareness, Care & Treatment in Obesity Management - an International Observation (ACTION-IO). ClinicalTrials.gov: NCT03584191

**Level of evidence:**

Level V, cross-sectional descriptive study.

## Introduction

Obesity is a growing problem in most countries, including Italy. Based on the World Health Organization (WHO) definition, in 2015, 35.3% of Italian adults had overweight, and an additional 9.8% were affected by obesity [1]. Obesity is associated with an increased risk of morbidity [2–5] and mortality [4–8] because of the associated comorbidities, representing a critical public health concern. Furthermore, people with obesity (PwO) not only experience a decrease in health-related quality of life but also face stigma and discrimination, causing a negative impact on their emotional and mental well-being [9]. Besides its health-related effects, obesity also has a substantial socioeconomic impact. Several studies have demonstrated that obesity and the associated comorbidities give rise to increased direct and indirect healthcare costs, posing a significant burden on PwO [10].

Lifestyle interventions and behavioural therapy are recommended as first lines of treatment for PwO, with the addition of pharmacotherapy if these changes are insufficient to reach or maintain the recommended weight loss goal [11–14]. Bariatric surgery should be considered when the body mass index (BMI) is ≥ 40 kg/m^2^ or ≥ 35 kg/m^2^ accompanied by obesity-related complications. This option may also be considered for PwO with BMI ≥ 30 kg/m^2^ presenting with poorly controlled type 2 diabetes [11–14].

Despite the availability of evidence-based policy guidelines, the increasing prevalence of obesity suggests the poor implementation of these recommendations. In practice, suboptimal care is often provided to PwO [15–17], as demonstrated by the low rates of obesity diagnosis, documentation and management [18, 19], and inadequate knowledge of obesity treatment guidelines [20]. Healthcare professionals (HCPs) are uniquely positioned to identify, evaluate and manage obesity. However, studies have shown that few PwO report receiving weight-loss counselling, and of those, approximately one quarter have a follow-up appointment scheduled to review their weight [18, 20]. Studies also show that HCPs vary widely in their provision of pharmacological and/or surgical interventions [18, 21], suggesting a lack of familiarity with indications and patient qualifications for initiating treatment and/or referrals and misperceptions of the safety and/or efficacy of bariatric surgery and currently available weight loss medications [17, 22].

Barriers to effective communication between PwO and HCPs include uncertainty regarding patient and HCP roles for initiating weight discussions [22], the belief among PwO that it is their responsibility to manage their weight, HCPs’ discomfort with broaching the topic, fear of offending the patient, especially considering patients often have access to their medical records, and misperceptions regarding patient disinterest and motivation for losing weight [17–19]. Furthermore, a disconnect between HCPs’ confidence in their ability to manage patients with excess weight and the effectiveness of PwO in achieving weight loss goals has been documented [23]. To improve the access to quality obesity care, a better understanding of the disease and identification of the local barriers are required.

The Awareness, Care, and Treatment In Obesity maNagement-International Observation (ACTION–IO) study was conducted to identify the perceptions, attitudes, behaviours and barriers to effective obesity care across PwO and HCPs. The primary results from the global ACTION-IO dataset have been reported previously [24]. Here, we report the results from the ACTION-IO survey of PwO and HCPs in Italy.

## Methods

Methodology for the ACTION-IO study has been reported previously [24]. Briefly, the ACTION-IO study was a cross-sectional, non-interventional, descriptive study that collected data via an online survey conducted across 11 countries (Australia, Chile, Israel, Italy, Japan, Mexico, Saudi Arabia, South Korea, Spain, the United Arab Emirates, and the United Kingdom). Italian participants completed the survey between 4 June 2018 and 26 July 2018.

Eligible PwO were ≥ 18 years old and were residents of Italy, with a current BMI of ≥ 30 kg/m^2^ based on self-reported height and weight. The PwO sample was targeted for representative demographics based on age, gender, income and region. Eligible HCPs were medical practitioners who had been in practice for ≥ 2 years, were involved in direct patient care for ≥ 70% of their time and who had seen ≥ 100 patients during the past month, with ≥ 10 of these patients having a BMI ≥ 30 kg/m^2^. HCPs specialising in general, plastic or bariatric surgery were excluded.

All respondents provided electronic informed consent prior to the initiation of the screening questions and survey. In Italy, ethical approval was determined to be non-essential for a study of this nature, based on regulatory standards and precedent. The study complied with all laws and regulations regarding the management of personal information as required by Italy and the European General Data Protection Regulation. The study was conducted in accordance with the Guidelines for Good Pharmacoepidemiology Practices [25] and is registered with ClinicalTrials.gov, number NCT03584191.

Two questionnaires, one for PwO and one for HCPs, were developed by an international steering committee of obesity experts, in addition to three medical doctors employed by the study sponsor, Novo Nordisk. The questionnaires represented a modified version of the previous ACTION US [18] and ACTION Canada [26] studies. Question (Q) numbers provided in the figures refer to the respective survey question numbers. The questionnaires were published previously in the Supplementary Appendix of the global ACTION-IO study [24]. Five-point end-anchored Likert scales assessed agreement, where ‘1’ meant ‘do not agree at all,’ and ‘5’ meant ‘completely agree.’ Responses of 4 or 5 were coded and reported as ‘agree’ unless otherwise noted.

KJT Group (Honeoye Falls, NY, USA) conducted the online survey (Decipher Survey Software, FocusVision Worldwide Inc., Stamford, CT, USA) and managed the acquisition and analysis of data. Respondents were recruited through email and completed the survey in English or the native language of their country. Only data from those who completed the survey were included in the analyses. The final PwO sample was weighted to representative demographic targets. HCP data were not weighted. Data were summarised using descriptive statistics (means, medians, frequencies). Statistical significance testing was also conducted for relevant analyses within PwO or HCP respondent types using two-tailed Chi-square tests, *t* tests, or *z* tests and a significance threshold of *p* < 0.05. Adjustment for multiple testing was not undertaken as this research was exploratory and descriptive in nature. All statistical significance tests for PwO respondents incorporated weighting effects. Specifically, data were analysed for statistically significant differences for variables where we hypothesised a potential for confounding impact, such as gender for PwO and BMI for HCPs (among HCPs providing their height and weight).

## Results

A total of 1501 PwO and 302 HCPs completed the survey in Italy (Table 1), with response rates of 19% for PwO and 33% for HCPs and final eligibility rates of 9% and 66%, respectively. The mean completion time was 30 min for PwO and 37 min for HCPs. The majority of PwO (70%) presented with Class I obesity, and 12% were in the high-risk group (Class III). However, only 35% of PwO perceived their current weight as obese or extremely obese. Furthermore, 75% of PwO had at least one comorbidity. Of the Italian HCPs, 56% considered themselves an expert in obesity/weight loss management or worked in an obesity clinic. In addition, about half (51%) of HCPs reported receiving advanced formal training in obesity treatment.

**Table 1:** Sample demographics and characteristics

	PwO (*n* = 1501)	HCPs (*n* = 302)
Mean age, years (range)	47 (18–88)	55 (31–72)
Gender		
Male	679 (45%)	232 (77%)
Female	821 (55%)	70 (23%)
Other	1 (< 1%)	0 (0%)
BMI classification		
Respondents	1501 (100%)	259 (86%)
Underweight or healthy range (< 25 kg/m^2^)	0 (0%)	148 (57%)
Overweight (25–29.9 kg/m^2^)	0 (0%)	94 (36%)
Obesity class I (30–34.9 kg/m^2^)	1021 (70%)	10 (4%)
Obesity class II (35–39.9 kg/m^2^)	278 (17%)	2 (1%)
Obesity class III (≥ 40 kg/m^2^)	202 (12%)	5 (2%)
Number of comorbidities		
0	480 (25%)	
1	374 (24%)	
2	291 (22%)	
3	185 (15%)	
≥ 4	171 (14%)	
HCP category		
PCP		152 (50%)
Specialist		150 (50%)
Diabetologist/endocrinologist		26 (9%)
Cardiologist		80 (27%)
Internal medicine (non-PCP)		27 (9%)
Nutritionist		3 (1%)
Other		14 (5%)
Obesity specialist^a^		
Yes		205 (68%)
No		97 (32%)

All N sizes for PwO are from unweighted data. Demographic percentages (age, gender) also are from unweighted data. All non-demographic percentage results are for PwO weighted data. HCP data was not weighted; therefore, N sizes and percentages are all unweighted data

*BMI* body mass index, *HCP* healthcare professional, *PCP* primary care physician, *PwO* people with obesity

^a^A physician who meets at least one of the following criteria: at least 50% of their patients are seen for obesity/weight management, or has advanced/formal training in the treatment of obesity/weight management beyond medical school, or considers themselves to be an expert in obesity/weight loss management, or works in an obesity service clinic

Both PwO (84%) and HCPs (77%) acknowledged the large impact of obesity on overall health. However, although 91% of HCPs perceived obesity as a chronic disease, fewer PwO (62%) shared this perception. Importantly, only 13% of PwO and 19% of HCPs agreed that society and/or the healthcare system in Italy was currently meeting the needs of PwO.

**Fig. 1:**
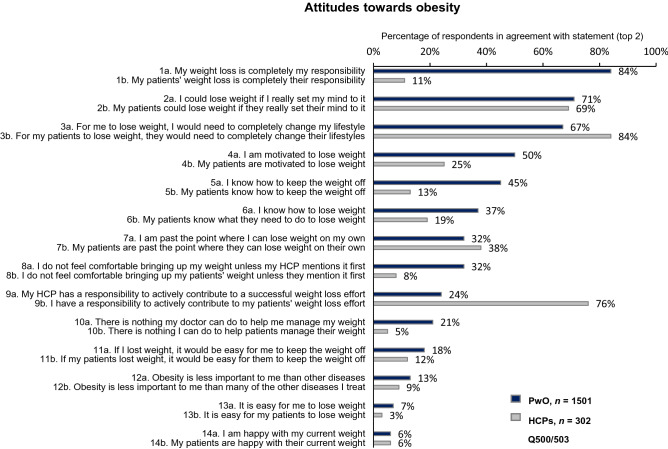
PwO and HCP agreement with statements regarding attitudes towards obesity, rated on a scale of 1–5. HCPs = grey; PwO = blue. *HCP* healthcare professional, *PwO* people with obesity

Most PwO (84%) assumed full responsibility for weight loss, despite acknowledging the impact of obesity on overall health (Fig. 1, item 1). The majority (67%) attributed their struggle with obesity to lifestyle, and only a few PwO (24%) felt that HCPs have a role in contributing to their weight loss efforts (Fig. 1, items 3, 9). Of the HCPs, 11% placed the responsibility of weight loss on PwO, and 84% agreed that PwO would need to change their lifestyle to lose weight. Most HCPs (76%) acknowledged their responsibility for their patients’ weight management.

Lack of exercise (72% PwO; 87% HCPs) and unhealthy eating habits (54% PwO; 83% HCPs) were considered as weight loss barriers by both groups. However, only a small proportion of PwO (37%) and HCPs (37%) regarded genetics as a contributing factor to obesity. Interestingly, compared with genetics, a higher proportion of PwO (60%) and HCPs (44%) perceived metabolism as a barrier to weight loss; HCPs with overweight or obesity themselves were significantly more likely than HCPs with normal weight to agree that metabolism represented a barrier (51% vs. 36%). Furthermore, 72% of HCPs believed the mental health and the emotional state of PwO to be barriers to weight loss, yet fewer PwO (41%) agreed with this statement. Additionally, compared with HCPs (58%), fewer PwO (18%) regarded their inadequate knowledge of obesity as a weight loss barrier. HCPs with overweight or obesity were significantly more likely than HCPs with normal weight to agree that PwO’s jobs or other health conditions are a barrier to PwO losing weight (45% vs. 31% and 54% vs. 41%, respectively).

Half of PwO reported that they were motivated to lose weight; however, only 25% of HCPs acknowledged their patients’ motivation for weight loss (Fig. 1, item 4). Indeed, the majority of PwO (85%) had undertaken at least one serious weight loss effort in the past, and when describing their level of concern with weight, only 20% reported that they had no plans of attempting weight loss within the next 6 months. On the other hand, HCPs acknowledged that, on average, only 38% of their patients with obesity had made a serious effort to lose weight, and they considered only 38% of these attempts as successful.

**Fig. 2:**
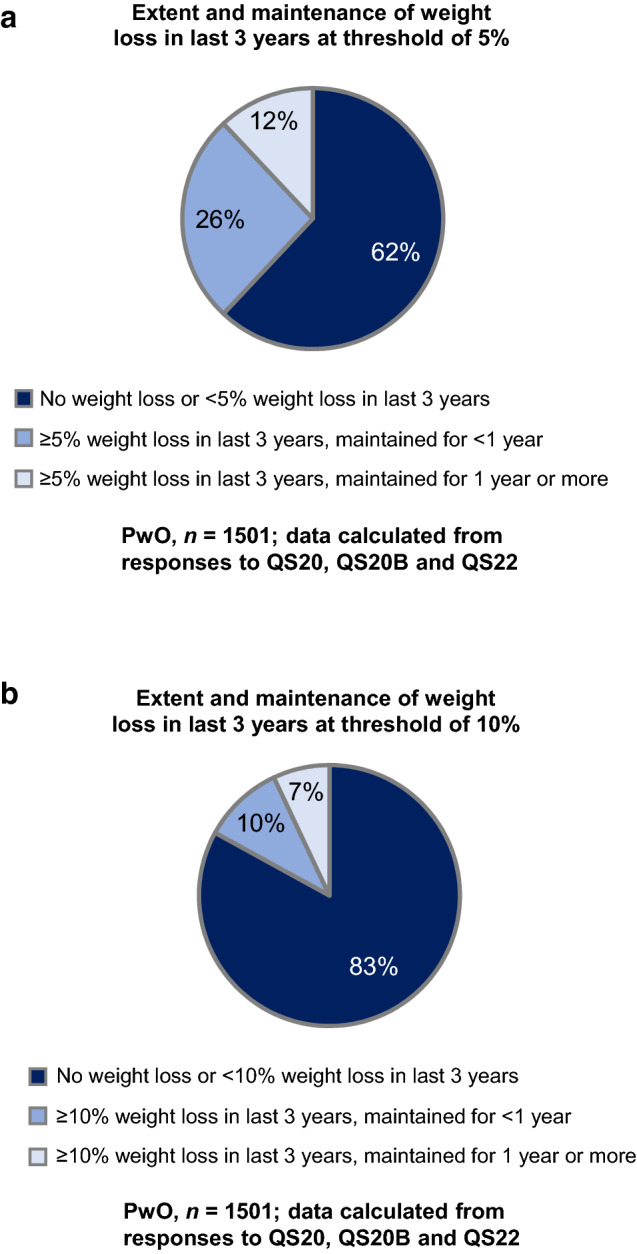
Weight loss attempts and maintenance. PwO extent and maintenance of weight loss in the past 3 years at a threshold of (**a**) 5% or (**b**) 10% of total body weight. *PwO* people with obesity

Regarding their attitudes towards weight loss, 71% of PwO believed that they could lose weight if they set their mind to it (Fig. 1, item 2). However, many PwO struggled to lose weight and maintain their weight loss (Fig. 2). Only 38% of PwO had a self-reported weight loss of at least 5% body weight over the past 3 years, and within this proportion, only 31% could maintain the weight loss for at least 1 year (12% of all PwO). Moreover, only 17% of PwO reported a body-weight loss of 10% or more over the past 3 years, and only 45% could maintain the weight loss for at least 1 year (7% of all PwO).

**Fig. 3:**
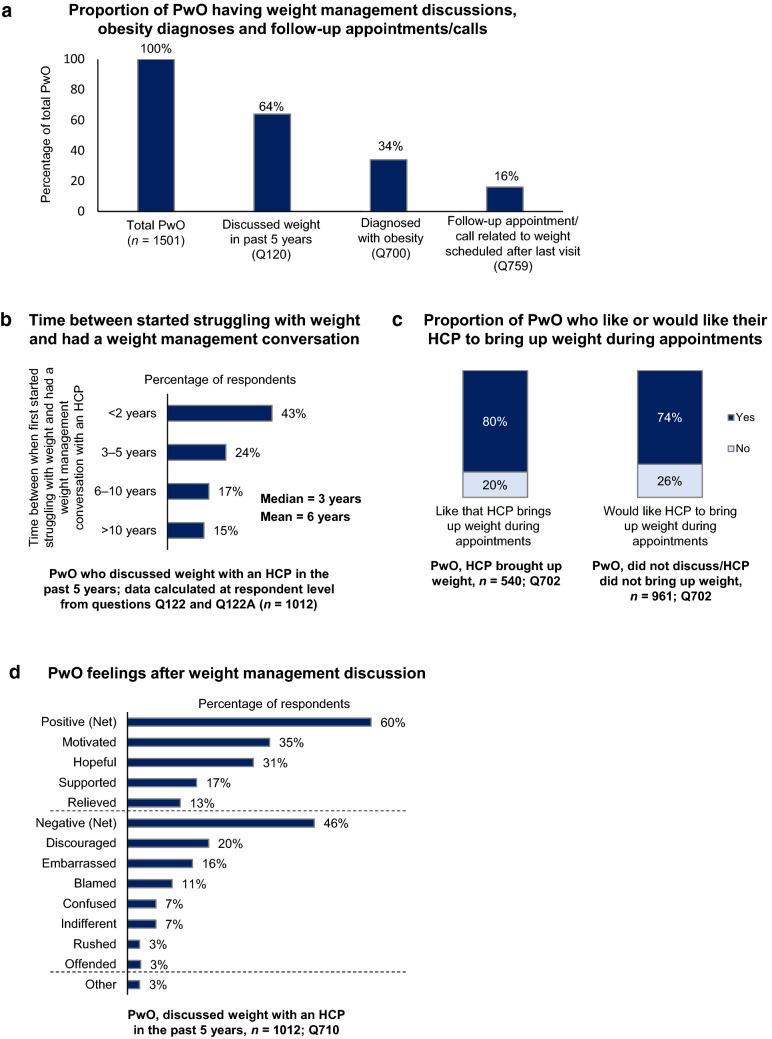
Attitudes towards weight management conversations. **a** Proportion of PwO having weight management discussions, obesity diagnoses, and follow-up appointments/calls with HCPs. **b** Time between first struggle with weight and having a weight management conversation with HCPs. **c** Proportion of PwO who like or would like their HCP to initiate weight discussions during appointments. **d** Feelings of PwO following weight discussions with an HCP. *HCP* healthcare professional, *PwO* people with obesity

In the past 5 years, 64% of all PwO had discussed their weight with an HCP (Fig. 3a). There was a median of 3 years and a mean of 6 years between the time PwO first struggled with weight and their first weight management discussion with HCPs (Fig. 3b). Among PwO who had a weight management discussion with HCPs in the past 5 years, 54% had been diagnosed with obesity (34% of PwO in total; Fig. 3a). HCPs reported informing 83% of their patients with obesity about this diagnosis; however, 6% of HCPs never informed their patients of a diagnosis of obesity. Of PwO who had a weight management discussion with HCPs, only 25% had a follow-up appointment scheduled (16% of PwO in total), although many PwO (94%) reported attending or planning to attend their follow-up appointment if scheduled. On the other hand, HCPs reported scheduling follow-up appointments for 44% of their patients with obesity, of which 78% reportedly kept these appointments always or most of the time.

When discussing weight management with HCPs, 45% of PwO initiated the conversation themselves. In contrast, HCPs reported that only 30% of their patients with obesity initiated these conversations. Obesity-related complications (76%), followed by patients’ BMI (65%), were the most commonly selected criteria by HCPs to initiate a weight loss discussion with their patients. Nevertheless, only 52% of HCPs expressed being extremely or very comfortable during these discussions.

When evaluating their weight management discussion with HCPs, 38% of PwO felt that these conversations were very helpful. Furthermore, most PwO expressed that they liked (80%) or would like (74%) their HCPs to raise the subject of weight during their appointments (Fig. 3c). More than one-third of the PwO who had discussed their weight with HCPs (35%) perceived these conversations as a motivating factor, and 60% reported positive feelings following the weight loss discussions. Negative feelings were reported by 46% of PwO, and only 3% felt offended by these discussions (Fig. 3d).

Among PwO who had not discussed their weight with HCPs, 38% believed that HCPs would be very helpful in finding solutions to assist with their weight loss efforts, while 4% believed that they would be not at all helpful. In addition, among PwO who had discussed their weight with HCPs, 25% reported that HCPs were very helpful in finding solutions to assist with their weight loss efforts, yet 11% felt that they were not at all helpful.

**Fig. 4:**
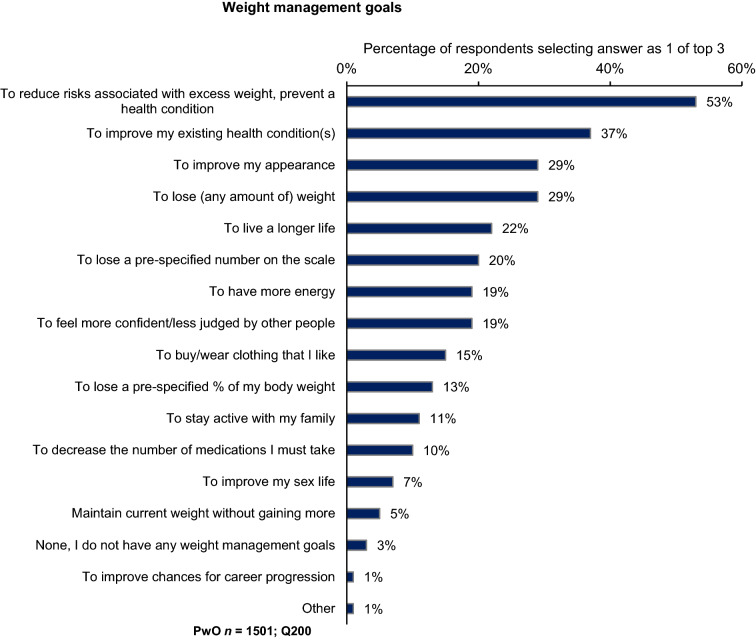
Most important weight management goals to personally achieve by PwO. *PwO* people with obesity

When deciding their goal weight, most PwO (75%) set themselves an ambitious target of > 10% body weight loss (overall mean 16.9%). PwO who had discussed their weight with HCPs reported receiving similar recommendations from HCPs, which would require, on average, an 18% body weight loss. For PwO, the most important weight management goals to personally achieve were reducing the risks associated with excess weight and preventing a health condition (53%), followed by improving existing health conditions (37%; Fig. 4). The most frequent weight management goal PwO set (35%) or would like to set (39%) with their HCPs was improving their lifestyle. Regarding the types of weight management goals HCPs set with PwO, 31% reported that they set short-term (next 6 months) individual weight loss goals; HCPs with overweight or obesity were significantly more likely than those with normal weight to set such goals with their patients (38% vs. 24%).

**Fig. 5:**
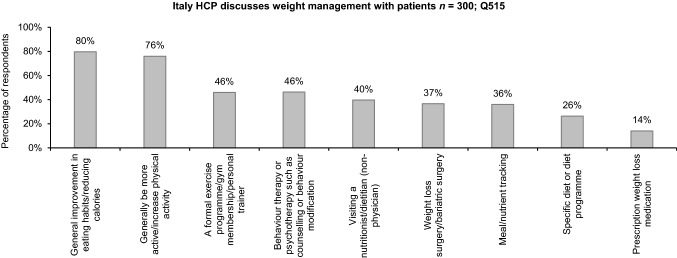
Effective long-term weight management recommendations by HCP. *HCP* healthcare professional

There was a common consensus between PwO and HCPs regarding the effectiveness of general improvements in eating habits (71% PwO; 80% HCPs) and physical activity (70% PwO; 76% HCPs) for weight management. Strikingly, a minority of HCPs perceived behavioural therapy, pharmacological treatment and bariatric surgery as effective weight loss methodologies (Fig. 5). Many PwO reported their preference for losing weight themselves rather than depending on medication (83%) or bariatric surgery (81%), while some HCPs believed that there are good options available today for weight loss medication (25%) or bariatric surgery (58%). HCP attitudes regarding weight loss surgery differed significantly among HCPs with overweight or obesity and HCPs with normal weight; HCPs with normal weight were significantly more likely than HCPs with overweight and obesity to agree that their patients trust them to recommend weight loss surgery if appropriate (62% vs. 48%), and that they are likely to review weight loss surgery options with their patients (58% vs. 44%, respectively).

The most frequent methods recommended by HCPs during weight management conversations with PwO were general improvements in eating habits/calorie intake (63%) and physical activity levels (63%). Other methods, including specific diet (22%) or exercise programmes (34%), prescription weight loss medications (11%) and bariatric surgery (10%), were less frequently considered during discussions. Referral to obesity specialists (24%) and nutritionists/dietitians (31%) were also infrequently recommended by HCPs.

**Fig. 6:**
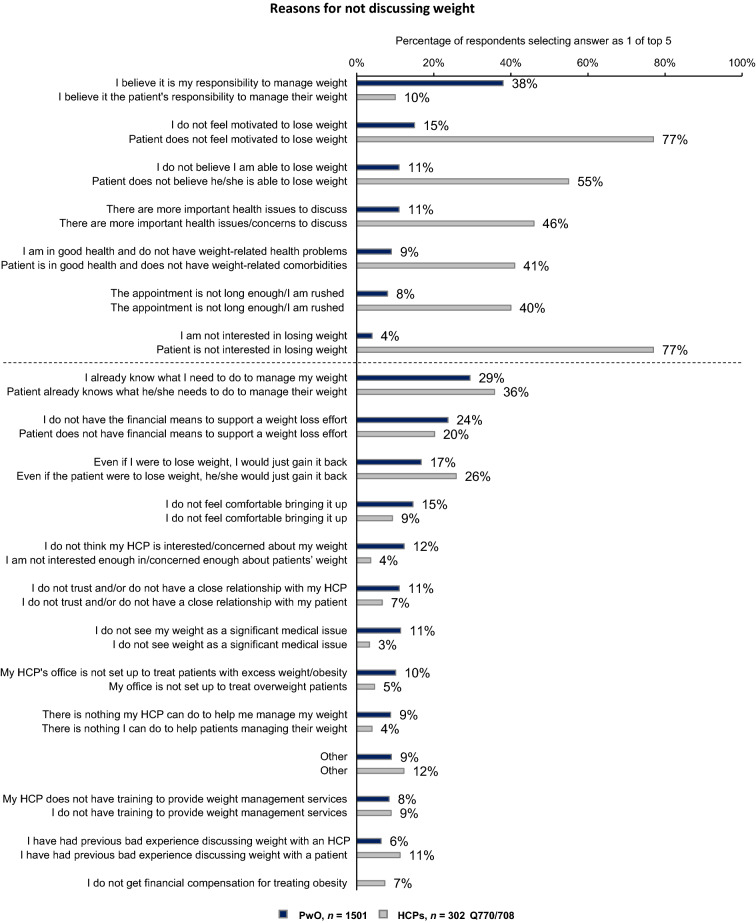
Reasons for not discussing weight with an HCP (PwO, blue) or patient (HCPs, grey); reasons with at least 10% difference between PwO and HCPs are above the dotted line, all others are below the dotted line. Respondents selected their top 5 reasons from the list of options. *HCP* healthcare professional, *PwO* people with obesity

For PwO, the top reason for not discussing weight management with HCPs was their belief that it was completely their responsibility to lose weight (selected by 38% of PwO vs. 10% of HCPs), with male PwO being significantly more likely than female PwO to cite this reason (46% vs. 30%). Male PwO were also significantly more likely than female PwO to indicate already knowing what they need to do to manage their weight and not seeing their weight as a significant medical issue as reasons for not discussing weight with their HCP (33% vs. 26% and 15% vs. 7%, respectively). Female PwO were significantly more likely than male PwO to not discuss weight with their HCP due to feelings of discomfort with broaching the subject (18% vs. 11%) and not believing they are able to lose weight (15% vs. 9%). On the other hand, the top reasons provided by HCPs for not engaging in weight loss conversations were perceiving their patients as disinterested (selected by 77% of HCPs vs. 4% of PwO; Fig. 6) or unmotivated (selected by 77% of HCPs vs. 15% of PwO) to lose weight. Among the top reasons HCPs gave for not discussing weight management with PwO was the belief that the patient does not believe he/she is able to lose weight (55%); HCPs with overweight or obesity were significantly more likely than HCPs with normal weight to cite this reason (63% vs. 50%). Finally, 40% of HCPs considered limited appointment time as a restricting factor for weight management discussions and/or they felt too rushed to initiate these conversations (Fig. 6).

## Discussion

Italy’s results from the ACTION-IO study demonstrated several gaps in terms of perceptions and attitudes of PwO and HCPs towards obesity and obesity care. Obesity is recognised as a chronic disease by a number of national and international organisations [12, 27–29]. Although the majority of PwO and HCPs acknowledged the large impact of obesity on overall health, fewer PwO than HCPs considered obesity as a chronic disease. The perception of PwO that obesity might not be a chronic disease could contribute to their assumption of full responsibility for their weight loss. Only 24% of PwO believed that HCPs have a role in their weight management, while 76% of HCPs acknowledged their responsibility.

Our results highlighted differences in the perception of weight loss barriers between PwO and HCPs. Although half of PwO reported being motivated for weight loss, and most had attempted at least one serious weight loss effort in the past, only 25% of HCPs acknowledged their patients’ motivation for weight loss. This lack of recognition of their patients’ efforts by HCPs may demotivate PwO from openly sharing their efforts. On the other hand, more HCPs thought their patients’ mental health and emotional state to be a barrier to weight loss than PwO did, which could hinder HCPs from approaching the issue of weight with their patients. Even though both groups identified lifestyle as a key weight loss barrier, few HCPs regarded biological factors, including genetics, as contributors to obesity, demonstrating a need for HCP education. Furthermore, while more than half of HCPs considered their patients’ inadequate knowledge of obesity as a drawback, only a few PwO recognised their lack of knowledge. Providing better education for PwO might improve their understanding of obesity and its related comorbidities, so they could be more active stakeholders in their weight loss.

Many PwO believed that they could lose weight if they set their mind to it; however, PwO struggled to lose weight and maintain weight loss, suggesting that they have a limited response on their own. In the past 5 years, only 64% of PwO discussed their weight with HCPs, and there was a mean 6-year time gap between these discussions and their first struggle with weight. This gap underlines communication problems as a barrier to effective obesity care. Decreasing this time gap by initiating timely and effective weight loss discussions would be an efficient strategy to reduce obesity and its related comorbidities, with a positive impact on the economic burden of the disease. That PwO may not recognise the need to reduce the excess weight until it has an impact on their health further supports the need for HCPs to have a proactive attitude, so they can raise the topic of weight prior to the occurrence of obesity-related complications.

Among PwO who had weight management discussions with an HCP, 45% initiated these conversations themselves. However, the majority of PwO liked or would like for HCPs to raise the topic of weight during their discussions. Although PwO reported mostly positive feelings following these conversations, only about half (52%) of HCPs felt very or extremely comfortable when discussing weight with their patients. The positive attitude of PwO towards weight discussions, as demonstrated by the study findings, should be a motivating factor for HCPs to be more actively involved in their patients’ weight management.

The top reason for HCPs not initiating weight management discussions was the perception that their patients were disinterested or unmotivated to lose weight, while for most PwO, assuming full responsibility to lose weight prevented them from initiating these discussions. In addition, male and female PwO reported different reasons for not initiating conversations with their HCPs, so it may be important for HCPs to recognise that gender differences amongst PwO exist. HCPs also regarded limited appointment time as a restricting factor for engaging in weight loss conversations with their patients. Importantly, few PwO (13%) and HCPs (19%) thought that society and the healthcare system in Italy are currently meeting the needs of PwO. Therefore, a systemic modification is required to lower the barriers for weight discussions and obesity care.

Interestingly, attitudes and perceptions amongst HCPs differed depending on the BMI classification of HCPs. Compared with HCPs with normal weight, HCPs with overweight or obesity were more likely to recognise the biological barriers to weight loss and more likely to report setting short-term weight loss goals. Conversely, HCPs with overweight or obesity were also more likely to cite the patients’ lack of belief in being able to lose weight as a reason for not initiating weight conversations, indicating that they might be incorrectly perceiving their patients as having feelings of hopelessness. Other studies have reported that HCPs with normal weight were more likely to initiate weight conversations with PwO [30] and document a diagnosis of obesity [31] compared with HCPs with overweight or obesity; at any BMI classification, when the HCP’s perception of the patient’s weight exceeded that of the HCP’s own weight, the HCP was more likely to initiate a discussion and record a diagnosis of obesity [30]. HCPs with overweight or obesity were more likely to feel confident prescribing weight loss medication and report helping their patients achieve successful weight loss efforts [30]. Together with our study, these data suggest that HCPs’ own perceptions and biases heavily influence their behaviour and further support the need for HCP education.

Both PwO and HCPs considered general improvements in eating habits and physical activity as the most effective methods for long-term weight management. A low proportion of HCPs recognised behavioural therapy and evidence-based treatments, such as medication and bariatric surgery, as effective methods, highlighting a need for better education and training of HCPs. Furthermore, only one in four HCPs recommended visiting an obesity specialist as a method for managing weight, further emphasising the lack of awareness regarding the need for multidisciplinary approaches. In addition, in terms of the perspective of PwO, a majority reported preferences for losing weight themselves rather than undergoing bariatric surgery or medication. This also points out an educational need for PwO to have a better understanding of the current treatment landscape of obesity to implement the most suitable treatment strategies best suited to their needs.

Overall, the findings from Italy are in line with those of the global cohort [24], with Italian PwO and HCPs having attitudes and beliefs similar to their global counterparts. This suggests that all countries share common problems in obesity care and the implementation of effective weight control strategies. To overcome these problems, several actions could be undertaken. Firstly, it would be important to improve the awareness of PwO, HCPs, governments and the public in terms of the biological basis of obesity to obtain unanimous recognition of obesity as a chronic progressive disease. Secondly, the misconception that obesity is under an individual’s control [32] should be challenged by addressing the attitudes of HCPs towards PwO and promoting earlier initiation of weight loss conversations. Thirdly, it would be necessary to improve the education and formal training of HCPs regarding the clinical management of obesity by emphasising the importance of a multidisciplinary approach [11–14]. HCPs should play a more proactive role in initiating weight management conversations with PwO, and increase the frequency of diagnosis, follow-up appointments and referrals for implementation of effective evidence-based treatments [33].

This study has several strengths and limitations. The strengths of the study include a large number of respondents and the sound methodological approach of the conducted survey, including stratified sampling to provide a representative cohort of the general population. Limitations of this study include its cross-sectional and descriptive nature, reliance on self-reported height and weight and accuracy of respondent recall. Low response rates could also contribute to study limitations by affecting the sample representativeness.

In conclusion, our data suggest that most PwO recognise the impact of excess weight on health and are making serious efforts to lose weight, but they have a limited response on their own. The motivation of PwO for weight loss and their positive feelings towards initiation of weight discussions should encourage HCPs to initiate timely and open weight management discussions. Setting realistic and achievable weight loss goals would also represent an important component of the obesity management plan, providing motivation for continued patient engagement. Our study also reveals a need for improved education of both PwO and HCPs concerning the biology behind obesity and its management, and for a systemic modification of the current healthcare system to meet the needs of PwO more effectively.

### What is already known on this subject?

In 2015, 45% of Italian adults had overweight or obesity. Despite the public health concern, attitudes and perceptions of PwO and HCPs towards obesity/obesity care are inadequately surveyed in Italy.

### What this study adds?

Our study found that HCPs should initiate timelier and open weight management discussions with PwO, there is an education gap, and the current healthcare system needs modification to meet PwO’s needs.
